# Well-controlled hypothyroidism does not affect the macronutrient composition of colostrum

**DOI:** 10.3389/fnut.2026.1921550

**Published:** 2026-10-29

**Authors:** Joanna Maria Jassem-Bobowicz, Bartosz Pastuszak, Ewa Magdalena Sokołowska, Izabela Drążkowska, Sylwia Narkowicz, Iwona Domżalska-Popadiuk

**Affiliations:** 1 Division of Neonatology, Medical University of Gdańsk, Gdańsk, Poland; 2 Human Milk Bank, Department of Neonatology, University Clinical Center, Gdańsk, Poland; 3 County Health Centre Ltd. – Dr. Jadwiga Obodzińska-Król Hospital, Malbork, Poland; 4 Division of Biopharmacy and Pharmacokinetics, Faculty of Pharmacy, Gdańsk, Poland

**Keywords:** colostrum, human milk, hypothyroidism, infant nutrition, milk composition, neonate

## Abstract

**Introduction:**

Maternal hypothyroidism is a common endocrine disorder occurring during pregnancy and has been associated with adverse maternal and neonatal outcomes. When untreated or poorly controlled, hypothyroidism may influence lactation and milk composition, although the effects of well-controlled hypothyroidism on human milk remain insufficiently characterised.

**Methods:**

This prospective cohort study included 120 postpartum women: 45 with well-controlled hypothyroidism treated with levothyroxine during pregnancy and 75 healthy controls. We collected colostrum samples within the first 5 days postpartum and analysed them using mid-infrared spectroscopy (MIRIS^®^) to determine macronutrient composition, including lipids, protein, carbohydrates, total solids, and energy content. We used the Mann–Whitney test for statistical analysis, with effect size assessed by Cliff’s delta. No *a priori* power analysis was conducted; therefore, interpret the results in light of the sample size and observed effect sizes.

**Results:**

No significant differences were observed between the hypothyroid and control groups in any measured component of milk composition, including lipids, crude and true protein, carbohydrates, total solids, or energy content (for all components, the *p* value > 0.05). Effect sizes were negligible across all variables. Additionally, there was no significant difference in neonatal outcomes and maternal characteristics between groups, indicating appropriate clinical management of hypothyroidism during pregnancy.

**Discussion:**

Well-controlled maternal hypothyroidism treated during pregnancy does not affect the macronutrient composition or energy content of colostrum in early lactation. The results indicate that adequate treatment of hypothyroidism does not affect the macronutrient composition or energy content of early colostrum. However, additional research to assess the composition of mature milk, the bio-active components and long term lactational outcomes must be done before wider implications for breastfeeding and human milk donation can be made.

## 1 Introduction

### 1.1 Definition and aetiology of hypothyroidism

The incidence of hypothyroidism has been steadily increasing in recent decades (1). The leading worldwide cause of hypothyroidism is iodine deficiency in the diet, followed by an autoimmune disease, Hashimoto’s thyroiditis. Less common causes include prior radioiodine ablation or surgical thyroidectomy, dysfunction of the hypothalamus and the anterior pituitary gland or congenital thyroid dysfunction (2). It is estimated that 3 to 5.5% of pregnant women suffer from hypothyroidism, making it the second most frequent obstetric endocrine disease (3). Given the importance of thyroid hormones for foetal and neonatal development, the accurate diagnosis of this condition is crucial. Thyroid hormones have a substantial impact on brain maturation, especially in its earliest stages, with subsequent effects on neonates. Consequently, untreated hypothyroidism during pregnancy may lead to adverse maternal outcomes, including gestational hypertension and preeclampsia, an increased risk of postpartum haemorrhage, placental abruption, and preterm delivery, which in turn increase perinatal morbidity and mortality (4, 5).

### 1.2 Composition of mother’s milk

Human milk is a complex biofluid that provides nutrients and bioactive components essential for infant growth and development (6). Its composition changes throughout lactation, with substantial differences between colostrum and mature milk (7). Maternal health, nutritional status, and lifestyle may further contribute to this variability. For example, maternal overweight and obesity have been associated with alterations in the fatty acid composition of human milk (8), while maternal diet and micronutrient intake may influence selected milk components. Therefore, macronutrient analysis reflects only one aspect of the complex and dynamic composition of human milk.

### 1.3 Impact of hypothyroidism on breast milk composition

Several maternal diseases, such as gestational diabetes and gestational hypertension have been associated with alterations in human milk composition (6, 7). Hypothyroidism as also been suggested to affect both milk production and composition (8). Previous studies have reported variable findings regarding human milk macronutrients, with some suggesting difference in protein, fat and carbohydrate concentrations compared with women with normal thyroid function (9). Maternal hypothyroidism has also been associated with alterations in milk lipid and fatty acid profiles, including polyunsaturated and trans fatty acids, even among women receiving thyroid hormone replacement therapy (10). Hypothyroidism may also affect lactation performance. Thyroid hormones interact with endocrine pathways involved in mammary gland development and lactogenesis, and inadequate thyroid function has been associated with delayed lactogenesis and reduced milk supply (8, 11–13). Although breastfeeding is considered safe during levothyroxine treatment, the evidence regarding lactation and human milk composition in women with well-controlled hypothyroidism remains limited (14, 15).

Available evidence on the relationship between maternal hypothyroidism and human milk composition remains limited and heterogeneous. Previous human studies have reported alterations in milk macronutrients and other physicochemical characteristics in association with maternal hypothyroidism (9). More recently, Alodhaybi et al. prospectively evaluated macronutrient and fatty acid composition in women with hypothyroidism; however, they collected milk samples at 4–13 weeks postpartum, representing a substantially later stage of lactation (10). In contrast, the present prospective study focuses specifically on early colostrum collected within the first 5 days postpartum in women with well-controlled hypothyroidism receiving levothyroxine treatment. This design allows assessment of whether adequately controlled maternal hypothyroidism is associated with alterations in macronutrient composition during the earliest stage of lactation.

## 2 Materials and methods

Inclusion criteria comprised women aged ≥18 years, singleton or multiple pregnancies, and availability of colostrum samples within the first 5 days postpartum. All of the participants were in-patient, post-partum mothers, who delivered a baby at the Medical University of Gdansk, Poland. In the hypothyroidism group, all patients received levothyroxine therapy during pregnancy and remained clinically euthyroid. Well-controlled hypothyroidism was defined based on thyroid-stimulating hormone (TSH), free thyroxine (fT4) and thyroid peroxidase antibodies levels within pregnancy-specific reference ranges, in accordance with current clinical guidelines of Polish Endocrine Society (16). We based the classification on thyroid function results documented in participants’ routine pregnancy medical records, obtained during standard obstetric and endocrinological care. We did not perform thyroid function testing at predefined study time points; instead, we followed routine clinical practise throughout pregnancy. The thyroid hormones were not routinely checked after the delivery in the first post-partum days. In the present analysis, the aetiology of hypothyroidism was not stratified and women with various underlying causes of hypothyroidism were analysed as a single group. We included thyroid autoantibody status for clinical classification but did not investigate it as a modifier of milk composition. Healthy controls were recruited post-partum if normal thyroid function was confirmed during the pregnancy based on the same criteria. In multiple pregnancies, only the first-born infant was included in the neonatal outcome analyses to ensure that all observations were independent. We offered no compensation for participation in the study.

Exclusion criteria were maternal smoking, pregestational or gestational diabetes, hyperthyroidism, history of breast surgery, using formula to feed the baby in the preceding feedings, and conditions potentially affecting milk composition.

We recruited patients between March 2024 and April 2026. The day of collection of colostrum was recorded in the first 5 days after the birth, but samples were analysed as a group as the number of samples on the individual postpartum days was too small. This should be taken into account when interpreting the findings, because of the dynamic changes in milk composition during early lactation. Larger samples are needed to examine time course of colostrum composition on selected number of days after parturition.

We asked every patient to provide a colostrum sample within the first 5 days postpartum. Although the protocol allowed colostrum collection during the first five postpartum days, most samples were obtained on days 4 or 5, when milk availability was greater. One sample was collected on day 2 and a small number on day 3. Mothers were generally approached regarding study participation on postpartum day 1 or 2, and sample collection was subsequently performed at a standardised time of day. The samples were collected between 8 a.m. and 2 p.m. to minimise circadian variability. After careful stirring, we cast up to 15 mL from a full expression volume from one breast. When possible, collection was standardised with respect to feeding timing (before the feeding) to reduce variability associated with foremilk and hindmilk composition. All mothers were instructed to follow this protocol; however, they were also informed that the infant’s feeding needs should always take precedence over the research protocol. Consequently, in some cases, milk expression may have been interrupted or preceded by infant feeding. We did not record adherence to this aspect of the sampling protocol.

To ensure participation in the study did not interfere with infant feeding, we collected milk samples only from mothers who had sufficient colostrum to meet their infant’s nutritional needs and could provide an additional sample for research purposes. Before sample collection, all participants were informed that their infant’s feeding requirements always took priority over study participation. Therefore, mothers experiencing insufficient milk production to adequately feed their infant in the early postpartum period were not asked to provide a research sample.

The milk samples were blinded immediately after collection by assigning anonymized study codes. The researchers performing the MIRIS analyses were blinded to the participants’ group allocation throughout the laboratory measurements. Access to the primary clinical data and group assignment was granted only after completing all milk analyses and unblinding the study codes.

Clinical data on the course of pregnancy, maternal demographic characteristics, anthropometrics and neonatal outcomes were gathered through direct questionnaire and from medical records (hospital records and outpatient pregnancy chart).

All patients expressed written informed consent. The study of maternal milk was granted the Medical University of Gdańsk Ethics Committee Approval no KB/449/2023.

### 2.1 Macronutrient content

All milk samples were aliquoted into 5 mL tubes and frozen at −20°C degrees immediately after acquisition. Before the analysis, the tubes containing milk were rewarmed to 40°C degrees, to ensure liquidity of fat. Next, homogenisation of each milk sample with an MIRIS ultrasonic processor (for 10 s) was performed immediately before the analysis. Then, the fat, crude protein, carbohydrate, total solids [g/100 mL], and energy [kcal/100 mL] were analysed using a human milk analyser (MIRIS^®^, Uppsala, Sweden) with mid-infrared spectroscopy. The MIRIS^®^ system quantifies certain macronutrients and energy content but cannot determine fatty acid composition, micronutrient content, oligosaccharides, hormones, immune factors, or bioactive proteins. However, the present study considers only part of the total composition of human milk. The present analysis could not take into account bioactive compounds, immune factors, micronutrients, fatty acid profile, mature milk composition. We handled and calibrated the MIRIS^®^ according to the manufacturer’s instructions. It was calibrated using the MIRIS Control Calibration Kit solutions at the beginning of each day and every ten cycles using the MIRIS Check Solution after. The milk samples were analysed twice or thrice if adequate milk volume was available. The samples were collected only from mothers who had surplus production of milk, and no harm to the feeding regimen of their own child was expected. Mothers were informed they were allowed to withdraw from the study at any time.

### 2.2 Statistical methods

The analysed dataset included macronutrient values from three consecutive measurements. To compare values between groups, we calculated the median of these three measurements for each component. We assessed normality using the Shapiro–Wilk test (*p* < 0.05). Due to the lack of normal distribution of the analysed variables, comparisons were performed using the Mann–Whitney test for continuous variables and Fisher’s exact test for binary variables. Additionally, we assessed effect sizes for between-group comparisons using Cliff’s delta and reported corresponding 95% confidence intervals. We estimated the 95% confidence intervals using bootstrap resampling with 30,000 iterations, performed separately within each study group. We did not perform an *a priori* sample size calculation. To aid interpretation of the null findings, a *post hoc* sensitivity analysis was performed to estimate the minimum standardised between-group difference detectable with the available sample size (*n* = 45 and *n* = 75), assuming a two-sided α of 0.05 and 80% power. The available sample size provided approximately 80% power to detect a standardised between-group difference of approximately 0.53, corresponding to a moderate effect size. Smaller differences therefore cannot be reliably excluded. The analysis of the association between gestational age and carbohydrate concentration was exploratory and was not based on a predefined hypothesis.

## 3 Results

The study included 120 postpartum women, 45 with well-controlled hypothyroidism and 75 healthy controls and their newborn children. Seven out of 120 pregnancies were multiple. Among the 120 newborns, there were 55 girls and 65 boys; 54 infants were born by cesarean and 66 born by spontaneous labour, 37 neonates were born preterm. Table 1 presents the characteristics of the study and control groups.

**Table 1:** Patients’ characteristics.

Characteristic	Patients diagnosed with hypothyroidism (*n* = 45)*	Healthy controls (*n* = 75)*	*P* value**
Maternal characteristics			
Maternal age (years)	31.8 ± 5.8	31.4 ± 5.0	0.56
Pre-pregnancy weight (kg)	65.1 ± 12.1	67.3 ± 17.0	0.84
BMI before pregnancy (kg/m^2^)	23.0 ± 3.9	24.0 ± 5.0	0.39
Gravidity	2 (1–2)	2 (1–3)	0.56
Parity	1 (1–2)	2 (1–2)	0.39
Multiple pregnancy (*n*)	1 (2%)	6 (8%)	0.25
Neonatal characteristics			
Male/Female sex of the neonate (*n*)	22/23 (49%)	43/32 (57%)	0.45
Gestational age (days)	269 (258–279)	267 (246–276)	0.27
Birth weight (g)	3,080 (2590–3,420)	3,010 (2315–3,495)	0.61
Head circumference (cm)	34 (32–35)	33 (32–35)	0.26
Body length (cm)	53 (51–56)	53 (49–55)	0.44
Apgar score	10 (9–10)	10 (9–10)	0.42

* Data were presented as number - *n* (%), mean ± standard deviation (SD), or median (interquartile range [IQR]), as appropriate. ** Comparisons were performed using the Mann–Whitney test for continuous variables and Fisher’s exact test for categorical variables.

Due to the lack of normality in the distributions of the analysed variables, comparisons were performed using the Mann–Whitney test for continuous variables and Fisher’s exact test for binary variables.

We observed no significant differences between groups (*p* > 0.05).

Additionally, effect size was assessed using Cliff’s delta. The effect size was negligible, ranging from −0.11 to −0.07. As there was no *a priori* power analysis, the observed small effect sizes should be taken with a grain of salt. The results indicate that there was little effect difference between groups, but larger studies may be needed to reveal smaller clinically relevant differences.

In the hypothyroidism group, the doses of hormone replacement therapy were adjusted throughout the pregnancy. The average daily dose of levothyroxine before the delivery was 89 ug [range 25–235 ug]. The etiologies (autoimmune and non-autoimmune) of hypothyroidism were not analysed separately due to limited sample sizes. In three patients, perinatal dosing data were missing.

Macronutrient content was measured three times in 113 patients; in seven patients, analyses were performed twice because of insufficient milk. The median of lipids, proteins, carbohydrates and energy content are presented in Table 2. Figure 1 represents box plots showing differences between the hypothyroidism and control group macronutrient content. The analyses shown in Table 2 and Figure 1 are limited to macronutrient composition and energy content and do not include fatty acid profiles, micronutrients, oligosaccharides, hormones, immune factors, or bioactive proteins.

**Table 2:** Analysis of milk composition in mothers diagnosed with hypothyroidism and healthy controls.

Macronutrient	Patients diagnosed with hypothyroidism (*n* = 45)*	Healthy controls (*n* = 75)*	*P* value**	Cliff’s Delta (95% CI)
Lipids (g/100 mL)	2.5 (1.8–2.9)	2.6 (2.1–3.2)	0.32	−0.11 (−0.32 to 0.1)
Crude protein (g/100 ml)	2 (1.9–2.1)	2.1 (1.9–2.2)	0.33	−0.11 (−0.31 to 0.11)
Carbohydrates (g/100 ml)	7.9 (7.6–8)	7.8 (7.6–8.1)	0.55	−0.07 (−0.27 to 0.15)
Total solids (g/100 ml)	12.6 (11.7–13.2)	12.6 (12–13.3)	0.42	−0.09 (−0.3 to 0.13)
Energy (kcal/100 ml)	64 (56–68)	63 (58.5–70)	0.40	−0.09 (−0.3 to 0.12)
True protein (g/100 ml)	1.6 (1.5–1.7)	1.6 (1.5–1.8)	0.39	−0.09 (−0.31 to 0.12)

* Data are presented as median (IQR). No significant statistical differences were found among groups for any of the measurements. The variability in the measured outcomes could, however, have been attributed to temporal variation in the composition of the colostrum over postpartum days 1–5 when samples were taken. ** Comparisons were performed using the Mann–Whitney test.

**Figure 1:**
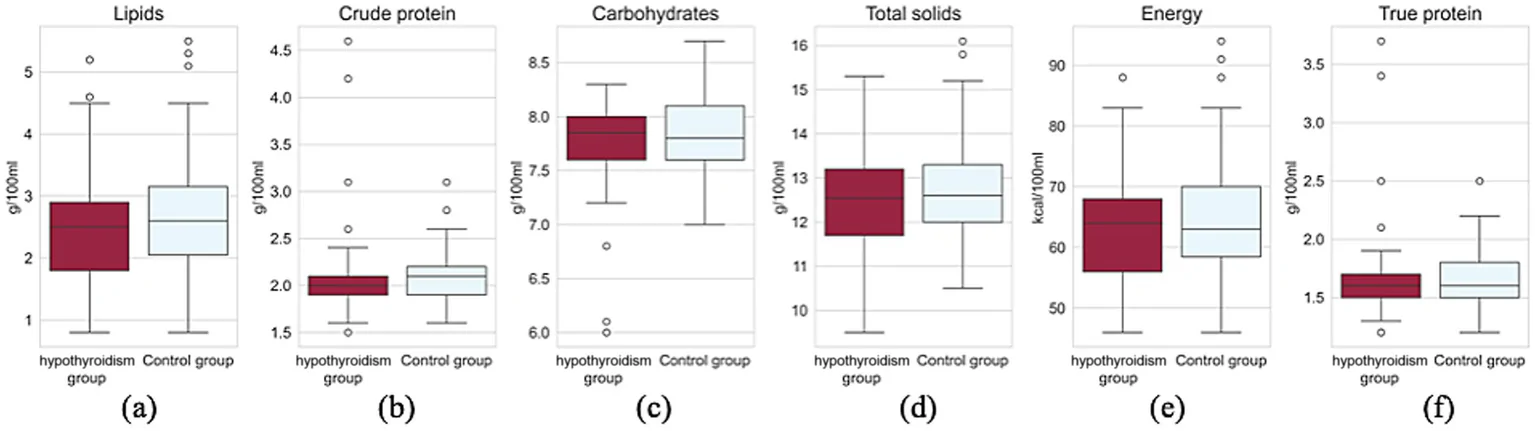
Box plots of macronutrient and energy content in the hypothyroidism (HT) group and the control group. **(a)** Lipids content in hypothyroidism group and healthy controls, **(b)** crude protein content in hypothyroidism group and healthy controls, **(c)** carbohydrates content in hypothyroidism group and healthy controls, **(d)** total solids content in hypothyroidism group and healthy controls, **(e)** energy content in hypothyroidism group and healthy controls, **(f)** true protein content in hypothyroidism group and healthy controls. Only macronutrient composition and energy content from MIRIS^®^ is presented and not other constituents of human milk.

Due to lack of differences between well-controlled hypothyroidism group (HT group) and healthy controls, further analyses were performed within the HT group. The association between the dose of levothyroxine and macronutrient content was investigated. Table 3 presents the results.

**Table 3:** Kendall correlation values between macronutrient values and levothyroxine dosage in the hypothyroidism group.

Hypothyroidism group	Kendall – levothyroxine dosage (μg)	
Lipids (g/100 mL)	Tau	0.10
	*P* value	0.37
Crude protein (g/100 ml)	Tau	0.02
	*P* value	0.84
Carbohydrates (g/100 ml)	Tau	0.04
	*P* value	0.73
Total solids (g/100 ml)	Tau	0.04
	*P* value	0.71
Energy (g/100 ml)	Tau	0.09
	*P* value	0.43
True protein (g/100 ml)	Tau	0.01
	*P* value	0.98

No statistically significant associations were observed between macronutrient values and levothyroxine dosages in the Hypothyroidism group (*p* > 0.05). Kendall’s tau correlation coefficient indicated weak positive association ranging from 0.01 to 0.10.

Figure 2 presents scatter plots with fitted regression lines. It is worth noting that fitting a linear regression line can be sensitive to outliers.

**Figure 2:**
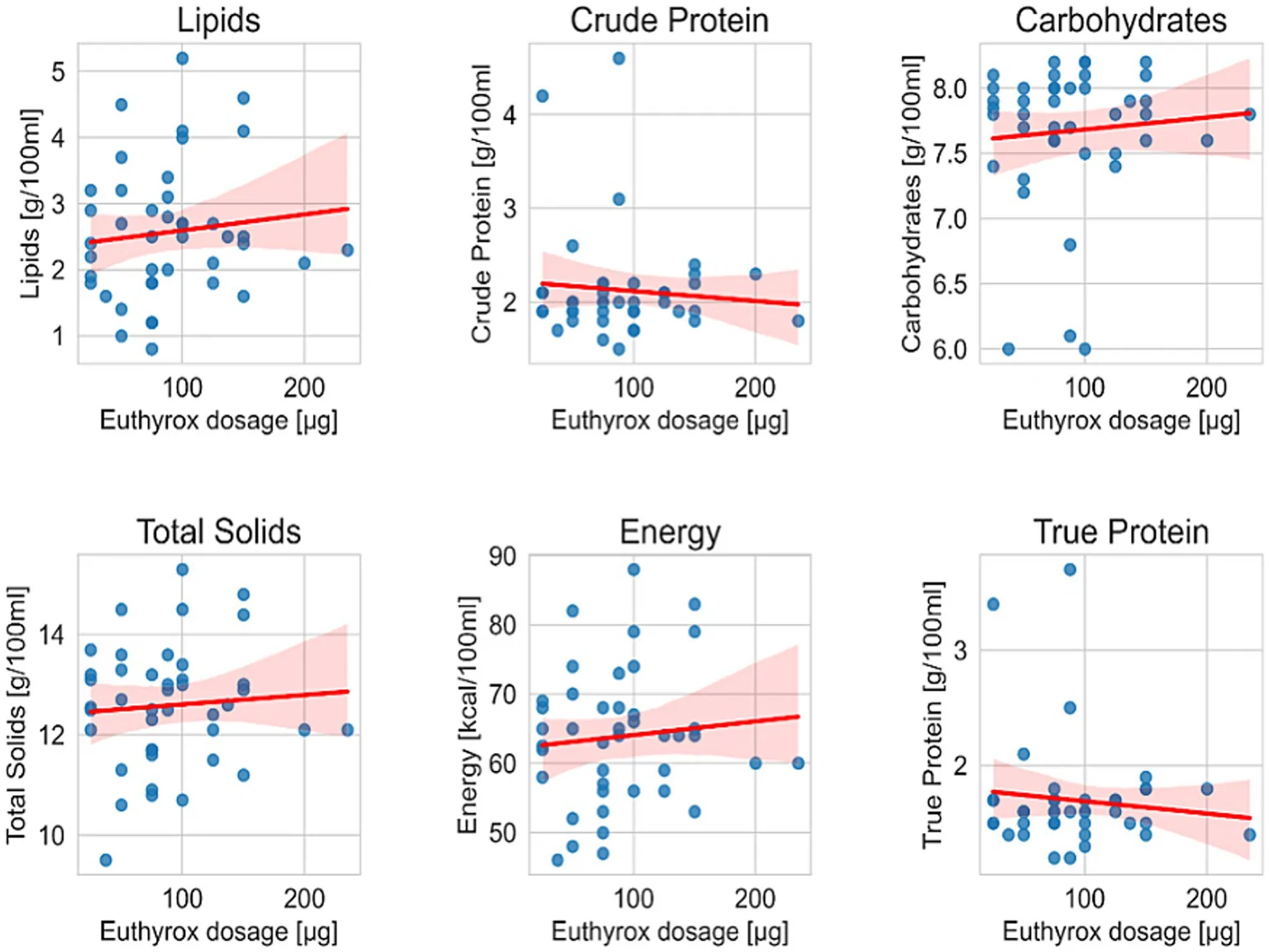
Associations between levothyroxine dose and colostrum macronutrient concentrations. Fitted linear regression lines are shown solely to facilitate visualisation of the direction of the relationships between variables and were not used for statistical inference. Statistical associations were assessed using Kendall’s tau correlation coefficient, as described in the Statistical Analysis section.

The analysis also examined how macronutrient composition in hypothyroidism and the healthy control group changed with gestational age. Because of small numbers at each postpartum day of milk collection, no stratified analyses were performed. A future study should assess changes in milk composition over time in early lactation. Protein, lipids, and energy content did not differ. However, carbohydrates remained constant in the HT group, whereas in the healthy controls, they corresponded with gestational age. These results are presented in Figure 3.

**Figure 3:**
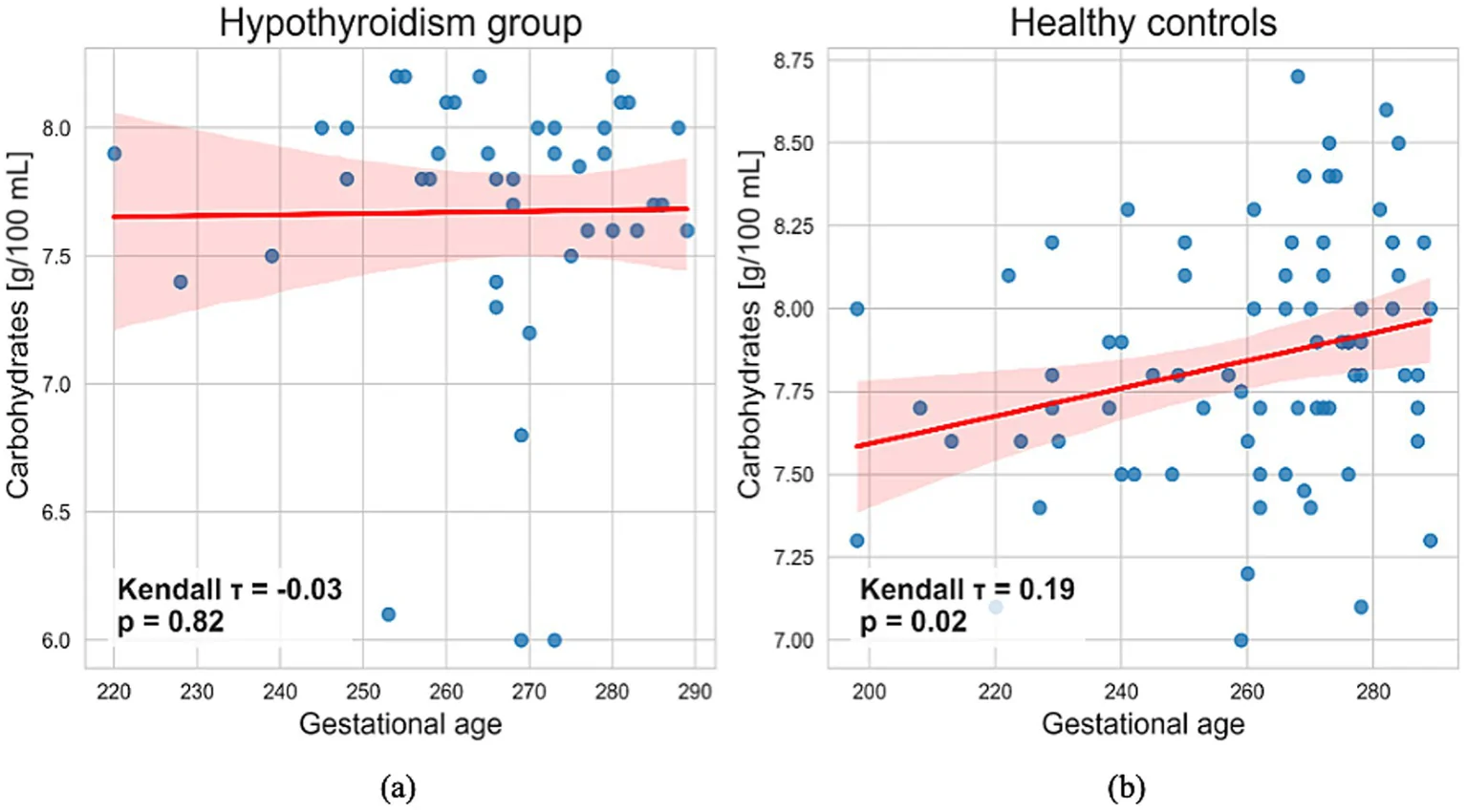
**(a)** Association between gestational age and colostrum carbohydrate concentration in **(a)** the hypothyroidism group (*n* = 45; gestational-age range: 220–289 days) and **(b)** healthy controls (*n* = 75; gestational-age range: 198–289 days). Gestational age is presented in days. Results can only be applied to early colostrum (1st to 5th postpartum days) and may not be applicable to mature milk.

## 4 Discussion

The primary objective of this study was to evaluate whether well-controlled maternal hypothyroidism treatment was associated with the macronutrient composition and energy content of human milk in the first 5 days post-partum. We performed an exploratory analysis to assess associations between levothyroxine dose and colostrum macronutrient concentrations. Because levothyroxine dose reflects individual replacement needs and clinical management rather than maternal thyroid hormone exposure, interpret these analyses cautiously. The results suggest no significant difference in the macronutrient composition and energy content of early colostrum between well-controlled hypothyroid and healthy controls.

The novelty of this study lies in the exclusive inclusion of women with well-controlled hypothyroidism and the focus on early lactation (colostrum), which has been insufficiently explored in previous studies.

From a nutritional perspective, the stability of macronutrient composition observed in this study is particularly important. Macronutrients in human milk are the primary determinants of neonatal energy intake and growth, especially during the early postnatal period. The absence of significant differences in lipid, protein, and carbohydrate content suggests that infants born to mothers with well-controlled hypothyroidism receive nutritionally adequate milk comparable to that of healthy mothers. The results herein are reassuring to women with well-controlled maternal hypothyroidism, although the present study was not specifically designed to assess breastfeeding outcomes, lactational performance or breastfeeding safety. Although all Cliff’s delta values were consistently negative, suggesting slightly lower values in the hypothyroidism group, the effect sizes were negligible and no statistically significant differences were observed. Consequently, these findings should be interpreted cautiously and are more likely to reflect random variation than a true biological effect; however, larger studies may clarify whether this consistent pattern reflects a subtle underlying trend.

Despite the established role of thyroid hormones in metabolism and lactation, lipid and protein concentrations were similar between groups. Milk lipid content was 2.5 g/100 mL in the hypothyroidism group and 2.6 g/100 mL in controls (*p* = 0.32), with no significant differences in crude or true protein. Previous experimental and clinical studies have reported reduced milk lipid and protein concentrations in uncontrolled hypothyroidism (17–19), whereas our findings suggest that these differences may not be apparent in women with well-controlled disease. However, total lipid concentration does not reflect lipid quality. Alodhaybi et al. reported differences in milk fatty acid composition in women with hypothyroidism, highlighting that similar total lipid concentrations do not exclude alterations in fatty acid profiles (9). Because the present study did not assess fatty acid and phospholipid composition, these differences cannot be excluded.

Exploratory analyses showed no significant associations between levothyroxine dose and colostrum macronutrient concentrations. However, levothyroxine dose reflects individual replacement requirements and clinical management rather than maternal thyroid hormone exposure and should therefore not be interpreted as a direct marker of thyroid status.

Carbohydrate concentration was not associated with gestational age in the hypothyroidism group, whereas a weak positive association was observed in controls. As this finding arose from an exploratory analysis without adjustment for multiple comparisons, it should be interpreted cautiously and requires confirmation. Carbohydrates are generally considered among the more stable components of human milk (20), although gestational-age-related differences in milk composition, including oligosaccharides, have previously been reported (21).

Neonatal characteristics and outcomes were similar between groups, including gestational age, birth weight, mode of delivery, and Apgar scores. This is consistent with adequate maternal disease control and is clinically relevant, as maternal thyroid dysfunction has been associated with adverse pregnancy outcomes, including an increased risk of small-for-gestational-age birth (22). However, the present study was not designed to assess the effects of hypothyroidism on pregnancy or neonatal outcomes, and these findings should therefore be interpreted cautiously. Similarly, the observed macronutrient composition of colostrum should not be extrapolated to breastfeeding performance or safety.

A key limitation is potential selection bias related to milk availability. For ethical reasons, we collected colostrum only when sufficient milk was available to meet the infant’s nutritional needs, as infant feeding was prioritised over study participation. Consequently, the study did not include mothers with delayed lactogenesis or insufficient milk production. This is particularly relevant in the context of hypothyroidism, which may be associated with impaired lactational performance. The study population may therefore represent women with relatively preserved milk production, potentially limiting the generalisability of our findings. Importantly, our results apply to the macronutrient composition of available colostrum and should not be interpreted as evidence that well-controlled hypothyroidism affects lactogenesis, milk volume, or overall breastfeeding performance. Although small colostrum volumes are physiological during the first postpartum days, our sampling approach may nevertheless have underrepresented women with particularly low milk production. Another important aspect is the fact that only colostrum was investigated in this study. Additional research on mature milk is warranted to draw further conclusions. In addition, we analysed macronutrient composition and energy content only using MIRIS^®^ technology, not fatty acid profiles, micronutrients, oligosaccharides, hormones, urine iodine, immune factors, or bioactive proteins. The collection window for the postpartum period (days 1–5) may also have contributed to the variation observed with rapid changes in colostrum composition.

For multiple pregnancies, we included only the first-born infant in the neonatal outcome analyses to maintain independence of observations. Consequently, the neonatal analyses do not reflect the full spectrum of outcomes among twins, which may limit the generalizability of these findings to multiple pregnancies.

We did not perform an *a priori* sample size calculation. A *post hoc* sensitivity analysis indicated that, with 45 women in the hypothyroidism group and 75 controls, the study had approximately 80% power to detect a standardised between-group difference of about 0.53 at a two-sided α of 0.05. Thus, the study was adequately powered to detect moderate or larger differences but cannot exclude smaller biological differences in colostrum composition. Accordingly, the absence of statistically significant between-group differences should not be interpreted as evidence of complete equivalence. Nevertheless, the consistently negligible observed effect sizes (Cliff’s δ, −0.07 to −0.11) suggest that any undetected differences are unlikely to be large.

In addition, we performed multiple statistical comparisons without adjustment for multiple testing. As this study was exploratory in nature, the findings should be interpreted with appropriate caution, particularly isolated statistically significant associations, which require confirmation in larger independent cohorts. Furthermore, we did not assess whether underlying etiologies of hypothyroidism or thyroid autoantibody status modified milk composition. Additionally, we did not systematically assess several maternal factors that may influence human milk composition, including maternal diet, iodine and selenium status, DHA supplementation, breastfeeding frequency, and postpartum hormonal status. As colostrum samples were collected during the short postpartum hospital stay, typically within the first few days after delivery, detailed data on these factors were not available. Consequently, their potential confounding effects could not be accounted for in the analyses. Future prospective studies should incorporate detailed nutritional, supplementation, lactation, and hormonal assessments to better control for these potential sources of variability. Future studies should use longitudinal sampling and more extensive compositional analyses.

## 5 Conclusion

In this prospective cohort, we observed no statistically significant differences in the measured macronutrient composition or energy content of colostrum collected during the first five postpartum days between women with well-controlled hypothyroidism receiving levothyroxine and euthyroid controls, and the between-group effect sizes were negligible. These findings are reassuring regarding the macronutrient composition of early colostrum in women with well-controlled hypothyroidism. However, they should not be interpreted as evidence of complete equivalence between groups or extrapolated beyond the outcomes investigated. The present study cannot exclude potential effects of hypothyroidism on fatty acid composition, micronutrients, immune factors, hormones, oligosaccharides, other bioactive components, mature milk composition, lactogenesis, milk production, or breastfeeding performance. Larger longitudinal studies incorporating broader compositional analyses and lactational outcomes are warranted.

## Data Availability

The raw data supporting the conclusions of this article will be made available by the authors, without undue reservation.

## References

[1] Wyne KL Nair L Schneiderman CP Pinsky B Antunez Flores O Guo D . Hypothyroidism prevalence in the United States: a retrospective study combining National Health and nutrition examination survey and claims data, 2009–2019. J Endocr Soc. (2022) 7:1–11. doi: 10.1210/jendso/bvac172, 36466005 PMC9706417

[2] Taylor PN Medici MM Hubalewska-Dydejczyk A Boelaert K. Hypothyroidism. Lancet. (2024) 404:1347–64. doi: 10.1016/S0140-6736(24)01614-3, 39368843

[3] Okosieme OE Lazarus JH. "Hypothyroidism in pregnancy". In: Feingold KR, Adler RA, Ahmed SF, Anawalt B, Blackman MR and Chrousos G, et al, editors. Endotext. South Dartmouth: MDText.com, Inc. (2000)

[4] The Consortium on Thyroid and Pregnancy—Study Group on Preterm Birth. Association of thyroid function test abnormalities and thyroid autoimmunity with preterm birth: a systematic review and meta-analysis. JAMA. (2019) 322:632–41. doi: 10.1001/jama.2019.10931 PMC6704759 31429897

[5] Singh S Haq N Sandhu S. "Thyroid disease and pregnancy". In: StatPearls. Treasure Island: StatPearls Publishing (2025) 30860720

[6] Sokołowska EM Jassem-Bobowicz JM Drążkowska I Świąder Z Domżalska-Popadiuk I. Gestational hypertension and human breast Milk composition in correlation with the assessment of fetal growth—a pilot study. Nutrients. (2023) 15:2404. doi: 10.3390/nu15102404, 37242287 PMC10222266

[7] Dugas C Laberee L Perron J St-Arnaud G Richard V Perreault V . Gestational diabetes mellitus, human Milk composition, and infant growth. Breastfeed Med. (2023) 18:14–22. doi: 10.1089/bfm.2022.0085, 36409543

[8] Lopes FDO Soares FVM Silva DAD Moreira MEL. Do thyroid diseases during pregnancy and lactation affect the nutritional composition of human Milk? Rev Bras Ginecol Obstet. (2020) 42:752–8. doi: 10.1055/s-0040-1718449, 33254271 PMC10309223

[9] Jin D Yu X Wang Q Chen X Xiao M Wang H . A study of the effect of hypothyroidism during pregnancy on human milk quality based on rheological properties. J Dairy Sci. (2024) 107:3400–12. doi: 10.3168/jds.2023-23900, 38135045

[10] Alodhaybi S Binobaed MA AlAnazi RH Elwehedy N Baraja M Aljawoan FY . Impact of maternal hypothyroidism on human milk macronutrient content and fatty acid composition: a prospective cohort study. Front Nutr. (2026) 13:1769344. doi: 10.3389/fnut.2026.1769344, 41743063 PMC12929449

[11] British Thyroid Foundation (2021). Breastfeeding and Thyroid Disease. Available online at: https://www.btf-thyroid.org/breastfeeding-and-thyroid-disease (accessed Apr 9, 2026).

[12] Osei J Andersson M Reijden OVD Dold S Smuts CM Baumgartner J. Breast-milk iodine concentrations, iodine status, and thyroid function of breastfed infants aged 2-4 months and their mothers residing in a south African township. J Clin Res Pediatr Endocrinol. (2016) 8:381–91. doi: 10.4274/jcrpe.2720, 27217155 PMC5197995

[13] Dror DK Allen LH. Iodine in human Milk: a systematic review. Adv Nutr. (2018) 9:347S–57S. doi: 10.1093/advances/nmy020, 29846524 PMC6008959

[14] La Leche League International. Breastfeeding and Thyroidism. Available online at: https://llli.org/breastfeeding-info/breastfeeding-and-thyroidism/ (accessed Apr 9, 2026).

[15] Levothyroxine. (2006). In: Drugs and Lactation Database (LactMed^®^). Bethesda: National Institute of Child Health and Human Development. Available online at: http://www.ncbi.nlm.nih.gov/books/NBK501003/ (accessed Apr 25, 2026).

[16] Hubalewska-Dydejczyk A. Trofimiuk-Müldner M. Ruchala M. Lewiński A. Bednarczuk T. Zgliczyński W. . (2021) Thyroid diseases in pregnancy: guidelines of the Polish Society of Endocrinology [Choroby tarczycy w ciąży: zalecenia postępowania Polskiego Towarzystwa Endokrynologicznego]. Endokrynol Pol. Available online at: https://journals.viamedica.pl/endokrynologia_polska/article/view/EP.a2021.0089/64652 (accessed Jun 7, 2026). 10.5603/EP.a2021.0089 34855189

[17] Capuco AV Connor EE Wood DL. Regulation of mammary gland sensitivity to thyroid hormones during the transition from pregnancy to lactation. Exp Biol Med (Maywood). (2008) 233:1309–14. doi: 10.3181/0803-RM-85, 18641053

[18] Neville MC McFadden TB Forsyth I. Hormonal regulation of mammary differentiation and Milk secretion. J Mammary Gland Biol Neoplasia. (2002) 7:49–66. doi: 10.1023/A:1015770423167, 12160086

[19] Tapia-Martínez J Franco-Colín M Ortiz-Butron R Pineda-Reynoso M Cano-Europa E. Hypothyroid offspring replacement with euthyroid wet nurses during lactation improves thyroid programming without modifying metabolic programming. Arch Endocrinol Metab. (2019) 63:199–207. doi: 10.20945/2359-3997000000132 PMC10522202 31066757

[20] Suwaydi MA Lai CT Rea A Gridneva Z Perrella SL Wlodek ME . Circadian variation in human Milk hormones and macronutrients. Nutrients. (2023) 15:3729. doi: 10.3390/nu15173729, 37686759 PMC10490050

[21] Sundekilde UK Downey E O’Mahony JA O’Shea CA Ryan CA Kelly AL . The effect of gestational and lactational age on the human milk metabolome. Nutrients. (2016) 8:304. doi: 10.3390/nu8050304 PMC4882716 27213440

[22] Derakhshan A Peeters RP Taylor PN Bliddal S Carty DM Meems M . Association of maternal thyroid function with birth weight: a systematic review and individual-participant data meta-analysis. Lancet Diabetes Endocrinol. (2020) 8:501–10. doi: 10.1016/S2213-8587(20)30061-9 PMC8168324 32445737

